# Effect of Slow-Release Urea Administration on Production Performance, Health Status, Diet Digestibility, and Environmental Sustainability in Lactating Dairy Cows

**DOI:** 10.3390/ani11082405

**Published:** 2021-08-14

**Authors:** Silvia Grossi, Riccardo Compiani, Luciana Rossi, Matteo Dell’Anno, Israel Castillo, Carlo Angelo Sgoifo Rossi

**Affiliations:** 1 Department of Health, Animal Science and Food Safety “Carlo Cantoni” (VESPA), Università degli Studi di Milano, 26900 Lodi, Italy; luciana.rossi@unimi.it (L.R.); matteo.dellanno@unimi.it (M.D.); carlo.sgoifo@unimi.it (C.A.S.R.); 2 Animal Science and Food Safety, University of Milan, 7, 20122 Milan, Italy; riccardo.compiani@gmail.com; 3 Phytotherapic Solutions, S.L.-Caldes de Montbui, 08140 Barcelona, Spain; israel@phytosolutions.es

**Keywords:** dairy, slow-release urea, efficiency, feed digestibility, sustainability, carbon footprint

## Abstract

**Simple Summary:**

The dairy system is facing many environmental issues, such as greenhouse gas emissions, land use, and consumption of human-edible raw materials, as well as increased demand for milk by the growing world population. Dairy cow farming must evolve toward more efficient and sustainable methods of production. Strategies to reduce the carbon footprint of the animal feed used and enhance overall productivity should be considered. Feed production, especially soybean meal, represents the second source of total dairy greenhouse gas emissions. Moreover, there is a positive correlation between production efficiency and environmental footprint. Using slow-releasing urea sources as an alternative to soybean meal can enhance rumen efficiency, functionality and reduce emissions related to the feed used due to a lower carbon footprint.

**Abstract:**

The effects of partially replacing soybean meal (SBM) with a slow-release urea source (SRU) on production performance, feed efficiency, digestibility, and environmental sustainability of dairy cows were evaluated. A total of 140 lactating Holstein Frisian cows were allocated into two study groups: (i) control (diet entirely based on SBM), and (ii) treatment (diet of 0.22% on dry matter basis (d.m.)) of SRU. Milk yield, dry matter intake (DMI), feed conversion rate (FCR), body condition score (BCS), reproductive parameters, and milk quality were evaluated. The chemical composition of the feeds and feces were analyzed to calculate the in vivo digestibility of the two diets. The carbon footprint (CFP) and predicted methane (CH_4_) emissions were evaluated. The inclusion of SRU significantly increases milk yield, DMI, and FCR (*p* < 0.0001), whereas milk quality, BCS, and reproductive indicators were not affected (*p* > 0.05). In the treatment group, the digestibility of crude protein (CP) (*p* = 0.012), NDF (*p* = 0.039), and cellulose (*p* = 0.033) was significantly higher, while the other nutritional parameters weren’t affected. All the environmental parameters were significantly improved in the treatment group (*p* < 0.0001). Replacing SBM with SRU can be a strategy to enhance dairy cows’ sustainability due to improved production efficiency, reduced feed CFP, and predicted CH_4_ production.

## 1. Introduction

The global population is expected to rise by 2 billion in the next three decades, increasing, in parallel, the demand for animal-derived products, with higher pressure on the food market to meet consumer requests [1,2]. In the past 60 years, this growth in the need for animal-derived foods has been met primarily by a steady increase in the number of animals reared and the nutritional value of the feeds, using higher levels of human-edible cereals and protein sources [3]. 

Those solutions are no longer feasible. In fact, zootechnical systems, especially dairy and beef cattle farming, confront many sustainability challenges, such as human-induced greenhouse gas (GHG) emissions, in which both animal rearing and feed production are involved [3,4].

In response to these concerns, more efficient and sustainable dairy production systems need to be developed. Strategies to reduce the ruminal CH_4_ production directly, enhance the overall production efficiency, and reduce the carbon footprint (CFP) of the feed used, may be considered. In fact, there is a positive relationship between production efficiency and environmental footprint, suggesting that strategies improving the productivity of dairy cows can lead to a simultaneous improvement in environmental impacts and profitability [4]. Moreover, a lower CFP of the diets is related to a lower emission intensity, with reduced emissions per unit of milk [5]. 

The use of alternative protein sources such as nonprotein nitrogen (NPN) to replace soybean meal (SBM) may be an effective strategy to address those challenges, mainly due to both the high environmental impact of SBM [6,7,8] and the positive effect of NPN at the ruminal level [9]. Traditionally, using alternative protein sources such as nonprotein nitrogen (NPN) to replace SBM was conducted primarily to reduce feed costs due to the high market prices of SBM and improve dietary protein utilization in dairy cows to enhance production efficiency [9]. In recent years, this strategy is gaining interest in mitigating the environmental impacts of dairy products and improving dairy cows’ productivity and efficiency [6,7,8]. 

Between the possible sources of NPN, feed grade urea was initially the most common in ruminants due to its low cost [10]. However, feed grade urea is characterized by rapid hydrolysis in the rumen, with a consequent fast release of ammonia, exceeding the rate of carbohydrate fermentation. Consequently, this condition reduces the production, flow, and availability of microbial protein for milk production and reduces the nitrogen (N) utilization efficiency [11]. Moreover, the rapid ruminal hydrolysis of urea increases N excretion through the urine and elevates blood NH_3_ levels, with a potentially negative effect on cattle fertility [12,13]. 

Coating technologies are used to develop slow-release urea (SRU) products for controlling the urea degradation rate and release of NH_3_ into the rumen, improving the efficiency of N utilization. The effects of SRU instead of SBM and feed grade urea have been reviewed in the literature, reporting positive effects on both beef [14,15] and dairy cattle [8,9,10,11,12,13,14,15,16,17]. Cherdthong et al. (2010) provided a narrative review of scientific literature that highlighted the potential efficacy of SRU in enhancing the efficiency of rumen N capture, microbial protein synthesis, and fiber digestion, with a consequent improvement in animals’ productivity and efficiency (cattle, buffalo, sheep, and goat) [18]. Specifically, in dairy cows, the inclusion of SRU instead of SBM or other traditional protein sources resulted in improved production performances, namely higher milk yield, increased feed efficiency, and improved feed conversion rate, as a result of a healthier, more stable, and efficient rumen [8,9,10,11,12,13,14,15,16,17]. 

A new slow-release urea source based on a matrix of urea prills covered by a two-layer lipidic stratification was recently developed (Protigen, Phytotherapic Solutions, S.L. 08140 Caldes de Montbui, Barcelona, Spain). More information about the product can be found in Supplementary Table S1.

We hypothesize that the partial substitution of soybean meal by the new sources of slow-release urea can be effectively used in dairy cattle due to its effect on rumen functionality, feed digestibility, production efficiency, and potentially lower environmental impact.

The present study aimed to evaluate the effects of the partial substitution of soybean meal (SBM) with a coated slow-release urea (SRU) source—Protigen—on the production performance, digestibility, and environmental impact, of high pedigree Holstein Frisian dairy cows.

## 2. Materials and Methods

### 2.1. Animal, Groups and Animal Care

The survey was conducted at the Del Santo farm located in Castelgerundo (Lodi, Italy), which well reflects the typical intensive dairy farm of the Po Valley area due to management and structural characteristics.

A total of 140 lactating Holstein Frisian cows were selected between the 200 lactating Holstein Frisian cows present on the farm at the beginning of the test and later enrolled in the trial. The animals were blocked by lactation number and days of lactation to create two balanced study groups with 70 cows each: (i) control (average lactation number of 2.30 ± 0.69; average days of lactation 53.86 ± 25.36); (ii) treatment (average lactation number of 2.31 ± 0.67; average days of lactation 51.86 ± 24.37). 

The animals were reared in two separate groups in the same free housing barn, on a concrete floor with straw-bedded cubicles. All the cows were milked twice a day, in the morning at 07:00 and in the evening at 17:00, in a herringbone milking parlor that allows the simultaneous milking of 16 cows (8 + 8).

The study lasted for 140 days.

### 2.2. Diets and Feeding Management

The two groups received two isoenergetic and isonitrogenous diets that differed for the protein sources used (Table 1). The control diet was based on soybean meal (SBM) as the main protein source and did not include any sources of slow-release urea (SRU). In the treatment diet, part of SBM (1.33% as fed, from 6.54 to 5.21%) was replaced, with 0.22% as fed (100 g/head/day) of SRU (Protigen). The SRU product used (Protigen) had a content of 250% of crude protein.

The two diets were formulated to meet or exceed the requirements for all nutrients [19].

The diets were administered ad libitum in the form of a total mixed ration (TMR) and distributed once a day in the morning through the use of a mixer wagon (Grizzly 71.26/2, capacity of 26 cubic meters mixing system with 2 vertical augers, Sgariboldi, Codogno, 2685 (LO), Italy), equipped with a balance, and designed to weigh both the inclusion of the individual ingredients and the unloaded TMR. Water was available ad libitum.

### 2.3. Parameters Recorded

#### 2.3.1. Production Performances: Milk Yield, Energy Corrected Milk (ECM), Milk Quality, Feed Intake, Feed Conversion Rate, Body Condition Score, Reproductive Performances

The daily milk yield (L/head/day) was recorded for each cow in the two groups. The milk yield was stored using a program, similar to the DairyComp programs available, developed specifically for the farm several years ago from a farm computer system company (Cremona, Italy). The feed intake for the two groups was evaluated daily by weighing the feed administered and then the residue in the manger 24 h later. The weekly average feed intake was calculated for both groups. The FCR was calculated, comparing the daily average feed intake with the daily average milk yield per group. Then the weekly FCR average was calculated for both groups. 

Milk quality analyses were performed monthly. Milk samples were analyzed for fat, protein, lactose, urea, and somatic cell counts. Milk analyses were performed by the Lombardy Regional Breeders Association (ARAL) laboratory with the Milkoscan TM FT 6500 Plus instrument (Foss, Hillerød, Denmark) that employs the Fourier Transform Infrared Spectroscopy (FTIR) measuring principle. The milk urea content was evaluated using a specific kit (Urea Assay Kit Rapid K-URAMR, Megazyme, Astori Tecnica s.n.c. Poncarale (BS) 25020). 

Monthly, the energy corrected milk (ECM) was evaluated by comparing the values of fat and protein obtained from the analyses and average milk production of the same week. The ECM was calculated following the equation proposed by Tyrrel and Reid (1965) [20]:

(1) ECM=0.327∗Milk yield (L)+12.95∗Fat yield(Kg)+7.2∗Protein Yield (kg)

The BCS was assessed monthly by the farm veterinary staff on all cows involved in the trial, as proposed by Edmonson et al. (1989) [21] and Ferguson et al. (1994) [22], through a visual and tactile evaluation of body fat reserves using a 5-point scale with 0.25-point increments (1—very thin cow; 5—excessively fat cow) where 3 represents the average body condition. The evaluation focused on the rump and loin. 

Reproductive performance was also evaluated in the two groups considering the days open and number of services for pregnancy as the main indicators of fertility.

All the cows were checked daily for health status by the farm veterinary staff.

#### 2.3.2. Characteristics of the Dies, Feces, and Digestibility of the Feeds

The characteristics of both the diet and feces were monitored twice per month (start and end of each month) using a portable NIR instrument (Polispec, IT Photonics, Fara Vicentino 36030 (VI), Italy). The monthly averages were then calculated. The characteristics of the TMR were analyzed in fresh feed with the portable NIR instrument while considering the entire bunk. Specifically, every time three measurements were gauged with the portable instrument along the entire length of the feed bunk (beginning, middle, and end of the manger). Similarly, the characteristics of the feces were analyzed for each group in a pool of fecal material collected the day after each feed analysis. The pool of fecal material was collected directly by a rectal grab in 20 cows per group. Samples of feces from the same group were then pooled together and mixed to create a single sample for each group. The pooled sample was analyzed with the portable NIR instrument. 

The portable NIR instrument directly analyzed the two substrates (feed and feces) for dry matter, crude protein, crude fats, acid detergent fiber (ADF), neutral detergent fiber (NDF), acid detergent lignin (ADL), starch, and ash. The content of hemicelluloses was obtained from the difference between NDF and ADF. The content of cellulose was obtained from the difference between ADF and ADL. Sugars and pectin were obtained by the calculation: 100 –(ash + fats + proteins + NDF + starch). 

The digestibility was evaluated through the following formula:

(2) $$Digestibility\;\%=\frac{\left(\frac{Xd}{ADLd}\right)-\left(\frac{Xf}{ADLf}\right)}{\left(\frac{Xd}{ADLd}\right)}\times 100$$

where:

*X* = each analytical parameter considered (%)
*ADL* = acid detergent lignin (%)
*d* = diet
*f* = feces.

#### 2.3.3. Environmental Impact: Diet Carbon Footprint (CFP)

The CFP of the two diets was calculated to evaluate the effect of partial replacement on the traditional SBM with an SRU source on greenhouse gas emissions.

The contribution of each feed’s raw material to the feed’s CFP was estimated by multiplying the inclusion level of the raw material and the CFP per kilogram of dry matter of raw material (g CO_2_-eq/kg). The CFP of each feed’s raw material was obtained from both the feed database created by Salami et al. (2021) [8], which includes CFP values from the Dutch FeedPrint and Plurimix software as well as the AgriFootprint databases (2014). The CFP for each raw material considers all the emissions derived from the field production, feed processing, and transport, including those derived from land-use changing (LUC). In order to quantify the CFP of the slow-release urea source Protigen, data derived from products with a similar composition, structure, and characteristics were used [8].

The average CFP of each TMR was then calculated and expressed as g CO_2_-eq.

The CFP of milk production as related to diet was calculated by dividing the weekly TMR CFP by the average weekly milk production. 

#### 2.3.4. Environmental Impact: Predicted Enteric Methane Production

Enteric methane production was estimated according to dry matter intake (DMI) using the equation of Hristov et al. (2013) [23], characterized by the highest coefficient of determination (R2) value (0.880; root mean square error: 15.3) between predicted and observed values [24], among all the possible equations available [25]. The equation is as follows:

*CH*_4_ (*g/d*) = *2.54* + *19.14* × *DMI* (3)

where:

1. *CH*_4_ = enteric methane production
2. *DMI* = dry matter intake (kg/head/day)

### 2.4. Statistical Analysis

Data analysis was conducted using SAS statistical software (SAS 9.4, SAS Institute Inc., Cary, NC, USA).

Data distribution and homogeneity of variances were tested using PROC UNIVARIATE (SAS 9.4, SAS Institute Inc., Cary, NC, USA). Data about production performance and environmental impact were analyzed using a mixed model (PROC MIXED), which considered the fixed effect of treatment and time of detection. For digestibility data of the single diet component, a residual estimate of maximum-likelihood was performed with PROC MIXED (SAS 9.4 SAS Institute Inc., Cary, NC, USA) on a mixed model considering the fixed effects of treatment, sampling day, their interaction, and the random effects of the animal within the treatment period. 

A single-subject was used as an experimental unit in all the statistical evaluations. 

For all the parameters, a *p*-value ≤ 0.05 was considered statistically significant, whereas a value ≤0.1 was considered a tendency.

## 3. Results and Discussion

### 3.1. Production Performances: Milk Yield, Energy Corrected Milk (ECM), Milk Quality, Feed Intake, Feed Conversion Rate, Body Condition Score, Reproductive Performances

**Figure 1:**
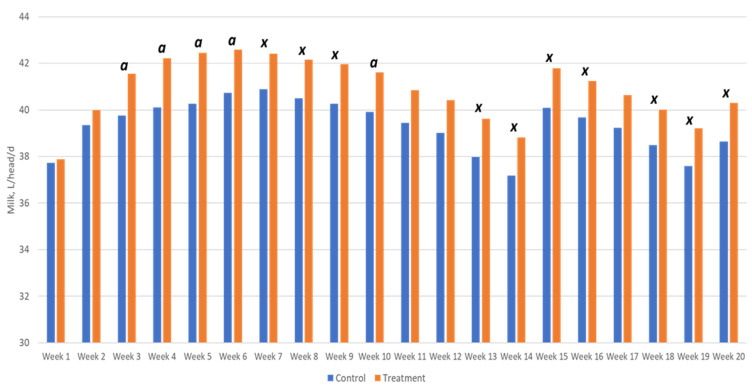
Average weekly milk production in the two groups (a = *p*-value ≤ 0.05; x = *p*-value ≤ 0.1).

Data about the production performance are reported in Table 2 and Table 3 and Figure 1. The partial substitution of SBM with an SRU significantly (*p* < 0.0001) improved the daily milk production and resulted in an average production increase of 3.9% during the entire trial period, corresponding to 1.54 L/head/day. Moreover, the results of ECM were also significantly higher in the treatment group (*p* = 0.0017). As shown in Figure 1, productivity began to differ between the two groups in the third week of the study, when the difference reached statistical significance. The literature also recognized that an integration of the diet aimed at influencing ruminal fermentation requires a period of at least 3 weeks to clearly show its effects [26]. As visible in Figure 1, milk production decreased from weeks 9 to 13 and increased sharply afterward. This great variation can be explained by changing environmental conditions (T °C and humidity). Firstly, between weeks 10 and 13, the adverse winter conditions, which were very cold with heavy rain and humidity, negatively affected both the animals and the microenvironment inside the stable. These conditions resulted in reduced feed intake and lower milk production with the declining health of the mammary gland. Conversely, from weeks 14 to 15, the environmental conditions improved quickly as spring began, resulting in better housing conditions (e.g., drier litter in the cubicles, cleaner and drier floors) and a more comfortable microenvironment inside the stable with positive reflexes on mammary health as well as feed intake and milk production. 

The result of the present study agreed with Tikofsky and Harrison (2007) [27] and Inostroza et al. (2010) [16], who reported a significant increase in milk production of cows fed diets containing SRU. Also, Kowalski et al. (2010) showed an improvement in milk production in high-yielding dairy cows fed with SRU in partial replacement of SBM [17]. Supplementation of SRU in ruminant diets fed with high levels of rapidly fermentable carbohydrates may increase the synchrony between the energy and protein availability at the rumen level, enhancing the microbial protein synthesis, thus improving its efficiency of converting into milk [28]. It should be emphasized that the use of urea (combined with enzyme and cereals as a slower and safer form of ruminally released nitrogen) dairy cow diets can beneficially modulate ruminal fermentation, including microbiota populations (an increase in relative abundances of *Megasphaera elsdenii* and ammonia-producing bacteria), consequently improving production performance as was mentioned by Libera et al., 2021 [29].

Conversely, Galo et al. (2003) [30], and Giallongo et al. (2015) [31], did not show any gain in milk production when SBM was partially replaced by SRU.

In the present study, DMI was significantly reduced in the treatment group (23.92 vs. 24.69 kg/head/d in control) (*p* = 0.04), positively affecting FCR. In fact, the FCR significantly improved (*p* < 0.0001) during treatment with an overall increase in feed efficiency at 6.9% due to the lower DMI and better milk production.

These results agree with the findings of Salami et al. (2021), who reported a 3% enhancement in feed efficiency due to a significant reduction in feed intake without any effects on milk yield when the traditional protein sources were replaced with SRU in dairy cows’ diets in Northern Europe [8]. 

Reproductive performance remained unaffected by the treatment (Table 2), which is in agreement with the findings of Hallajian et al. (2021), who reported similar characteristics of the follicles, blood levels of progesterone, and milk urea nitrogen (MUN) between dairy cows fed exclusively with SBM or with the partial replacement of SBM, SRU [32]. These results show that feeding with SRU appears to overcome the possible negative effect of other NPN sources, such as feed grade urea, on both plasma urea nitrogen and overall reproductive performance [33].

Body condition scores were not influenced by the treatment (Table 2). Similarly, Neal et al. (2014) [34] and Hallajian et al. (2021) [32] did not report significant differences in terms of body weight and body condition in dairy Holstein cows fed with diets containing SRU compared with SBM control diets. 

Also, the treatment did not influence milk quality traits, as reported in Table 3. These findings align with the main results found in the literature regarding dairy cows fed with an appropriate amount of slow-release urea [16,17,18,19,20,21,22,23,24,25,26,27,28,29,30]. 

No treatment effects were found in the overall health condition monitored daily by the farm veterinary staff.

The positive results observed after including SRU as a partial substitute for SBM underlined that ruminal kinetics and fermentation could be optimized in diets with a percentage of soluble protein higher than 30% of the total crude protein and 50% of degradable protein if combined with an adequate intake of nonstructural and rapidly fermentable carbohydrates.

Despite the significant increase in the solubility of the protein fraction, no changes in milk quality or reproductive performance were observed. Conversely, previous studies showed an increase in the milk urea levels and a reduction in fertility after an increase in protein solubility [35,36].

### 3.2. Characteristics of the Diets, Feces and Digestibility of the Feeds

Chemical characteristics of the diets are shown in Table 4 and Table 5, while chemical characteristics of feces are shown in Table 6 and Table 7. Results highlighted a good correspondence between the projection of the rationing software and the analytical characteristics found. 

Results for nutrients digestibility during the different months of the survey (Table 8) showed that the partial substitution of SBM with SRU significantly enhanced protein (*p* = 0.012), NDF (*p* = 0.039), and cellulose (*p* = 0.033) digestibility. Those results partially agreed with Sinclair et al. (2008), who reported a significant improvement in ruminal digestion of fiber in vitro [37]. These findings can be partially explained by an increased abundance and activity of fibrolytic bacteria in the rumen, such as Ruminococcaceae, which uses ammonia as its main nitrogen source due to both a higher level of NH_3_ availability and better synchrony between nitrogen and carbohydrates in the rumen, when SRU is used as a partial substitution for SBM [38,39]. Furthermore, Geron et al. (2016) found a better in vivo digestibility of crude protein in sheep fed with SRU as a partial replacement for SBM [40].

### 3.3. Environmental Impact: Carbon Footprint of the Feed (CFP) and Predicted Methane (CH_4_) Production

**Figure 2:**
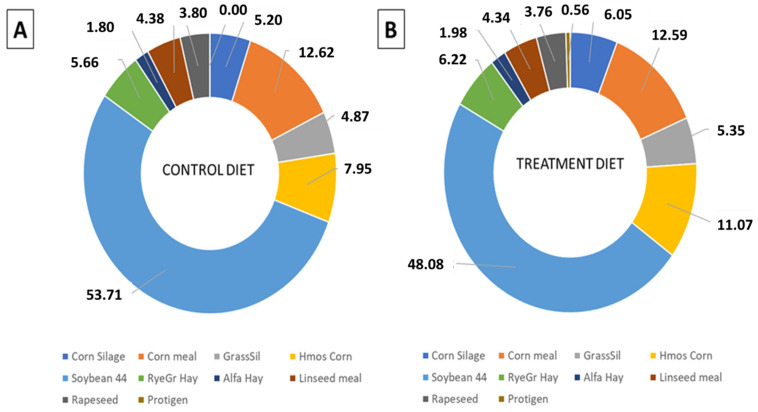
Contribution of different feeds’ raw materials to the average carbon footprint of (**A**) control diet, (**B**) treatment diet.

The impact of feeding SRU as a partial substitution for SBM on dairy sustainability was evaluated by estimating the CFP of the different diet feeds and predicting the enteric CH_4_ production (Table 9, Figure 2).

The results revealed that SBM was the dominant contributor to the feed CFP, accounting for 53.71% and 48.08% of the total CFP of the control and treatment diets, respectively (Figure 2). Notably, the inclusion of SRU in the treatment diet contributed only 0.56% of the feed CFP (Figure 2). These results align with the average values reported in other studies focused on intensive dairy cows farming, where SBM and other plant-protein sources were replaced by SRU [8].

The partial replacement of SBM with SRU decreased the CFP of the treatment diet, expressed per 1 kilogram of diet (−10.98%; 449.91 vs. 505.41 g CO_2_-eq/kg diet) compared to the control diet due to the lower global warming potential (GWP) of SRU than SBM [24]. The highest GWP of soybean meal is mainly due to large transport distances and emissions related to land-use change (LUC), such as deforestation, which can lead to heavier emissions of greenhouse gases [6,7,8,9,10,11,12,13,14,15,16,17,18,19,20,21,22,23,24,25,26,27,28,29,30,31,32,33,34,35,36,37,38,39,40,41]. These results agree with the findings of Salami et al. (2021), who showed a 12% reduction in CFP (g CO_2_-eq/kg diet) when SBM and other plant-protein sources were replaced by SRU [8].

The inclusion of SRU also significantly (*p* < 0.0001) reduced the CFP of the diet on a dry matter basis (10.76 vs. 12.48 kg CO_2_-eq/kg DMI) as a result of both the lower DMI and the lower CFP of the feeds (Table 9). Notably, the CFP of the control diet per total DMI aligned with the average values reported by Gislon et al. (2020) in a study conducted on 171 dairy herds in the same Po Valley area where the present study was conducted [24]. Conversely, the impact per kilogram of DMI in the treatment diet was lower than the range of values reported in that survey [24]. 

Similarly, the CFP of milk related to feed intake was significantly lower (*p* < 0.0001) in the treatment diet compared with the control diet (−16.89%; 266.40 vs. 320.53 g CO_2_-eq/L milk) as a result of both the higher daily milk production and lower CFP of the feeds (Table 9). The reduction of CFP in milk related to the feed intake found in the present study, which was also higher than the reduction (−14.5%) found by Salami et al. (2021) [8].

The partial substitution of SBM with SRU led to a significant (*p* < 0.0001) reduction in the predicted ruminal CH_4_ production (Table 9). It is important to underline that the present study did not quantify the real ruminal CH_4_ production. Instead, it was assessed by following the equation of Hristov et al. (2013) [23] based on dry matter intake (DMI). The lower CH_4_ production in the treatment group was a function of both the lower DMI when expressed only as grams of CH_4_ per day and better feed efficiency and milk yield when expressed as grams of CH_4_ per liter of milk.

Notably, there is limited published information on the effect of feeding urea on real enteric CH_4_ production. Alipour et al. (2020) [42], and Rebelo et al. (2019) [43] showed that feeding two sources of coated and non-coated urea did not affect enteric CH_4_ yield measured in an in vitro ruminal fermentation system or beef cattle, respectively. However, Libera et al. (2021) showed that the CH_4_ production at the ruminal level reduced after using urea combined with enzymes and cereals in dairy cows. This result was due to a reduction in the substrate available for methanogenesis and an influence on methanogens or other rumen microorganisms [29]. Although existing information suggests that slow-release urea (SRU) may have little or no effect on enteric CH_4_ emissions, there is a crucial need for future studies to address this lack in the literature.

## 4. Conclusions

This work contributes to defining scientific knowledge about the use of SRU in dairy cows’ nutrition and filling the present gap in the literature about their effect on environmental sustainability. 

This study showed that the substitution of traditional SBM (1.33% as fed) with the source of SRU Protigen (0.22% as fed, 100 g/head/d) led to an improvement in dairy cows’ efficiency due to an enhanced feed conversion rate, a lower dry matter intake, a higher digestibility of the fibrous parts of the diet and to better daily milk production. Furthermore, the present study showed that the environmental sustainability of dairy cows’ diets could be improved by including SRU as an alternative protein source, mainly reducing the impact of the feed production in terms of greenhouse gas emissions and predicted ruminal CH_4_ production. Further research is needed to evaluate the effective role of SRU on methanogenesis and real ruminal production of methane. 

## Figures and Tables

**Table 1:** Composition and nutritional values of the two diets tested, as predicted by the rationing software (Plurimix, Fabermatica, Ostiano (CR), Italy).

Feed, % as Fed	Control	Treatment
Corn Silage	50.56	52.33
Corn meal	11.39	10.12
Grass Silage	8.43	8.24
High-moisture Corn	6.74	8.35
Soybean meal 44% CP ^1^	6.54	5.21
RyeGrass Hay	5.84	5.72
Alfalfa Hay	4.20	4.11
Linseed meal	2.91	2.57
Rapeseed meal	1.82	1.60
Min Mix	1.57	1.54
Protigen	0.00	0.22
**Analysis, % of d.m. ^2^ in the TMR**		
d.m., %	54.81	53.77
Energy, Mcal/kg d.m.	1.62	1.62
CP ^1^	15.02	15.00
RDP ^3^, % on CP	62.69	65.33
RUP ^4^, % on CP	37.31	34.67
Sol CP ^5^, % on CP	28.83	34.40
Sol CP, % on RDP	45.98	52.66
Sugars	3.11	2.97
Starch	27.93	28.25
NDF	34.71	35.33
ADF	20.22	20.48
ADL	3.93	3.98
Fat	2.90	2.89
Ca total	0.84	0.85
p total	0.36	0.35

^1^ CP = crude protein; ^2^ d.m. = dry matter; ^3^ RDP = rumen degradable protein; ^4^ RUP = rumen undegradable protein. ^5^ Sol CP = soluble crude protein.

**Table 2:** Production performance: milk yield, feed intake, feed conversion rate, body condition scores, and reproductive performance in the two groups.

	Group		SEM	*p*-Value
	Control	Treatment		
**Production Performance**				
Milk yield, L/head/day	39.34	40.89	0.13	<0.0001
ECM ^2^, kg	43.20	44.87	0.37	0.0017
DMI ^3^, kg/head/day	24.69	23.92	0.04	<0.0001
FCR ^4^	1.59	1.70	0.004	<0.0001
**BCS ^4^**				
December, week 3	2.87	2.911	0.03	0.351
January, week 7	3.02	3.06	0.03	0.193
February, week 12	3.17	3.18	0.03	0.852
March, week 17	3.16	3.12	0.03	0.181
**Reproductive Performance**				
Days open	101.46	100.10	1.28	0.454
Services to pregnancy	2.08	1.97	0.09	0.402

^2^ ECM = energy corrected milk; ^3^ DMI = dry matter intake; ^4^ FCR = feed conversion rate; ^5^ BCS = body condition score.

**Table 3:** Production performance: milk quality analyses.

	December, Week 3	January, Week 7	February, Week 12	March, Week 17
**Fat, %**				
Control	3.81	3.82	3.84	3.84
Treatment	3.84	3.80	3.85	3.86
*p*-value	0.720	0.786	0.901	0.864
**Proteins, %**				
Control	3.67	3.69	3.67	3.65
Treatment	3.74	3.66	3.65	3.66
*p*-value	0.234	0.535	0.847	0.852
**Urea, mg/100 mL (mmol/L)**				
Control	21.45 (3.560)	23.13 (3.839)	22.28 (3.698)	22.29 (3.700)
Treatment	21.01 (3.487)	23.56 (3.910)	22.31 (3.703)	22.96 (3.811)
*p*-value	0.471	0.479	0.486	0.274
**Lactose, %**				
Control	4.82	4.83	4.84	4.82
Treatment	4.83	4.82	4.84	4.86
*p*-value	0.533	0.61	0.886	0.061
**Somatic cells, x.000**				
Control	426.57	488.79	496.29	470.56
Treatment	421.02	480.53	488.78	463.42
*p*-value	0.957	0.936	0.942	0.945
**Fat yield, kg/day**				
Control	1.517	1.563	1.497	1.510
Treatment	1.590	1.609	1.552	1.569
*p*-value	0.104	0.300	0.224	0.190
**Protein yield, kg/day**				
Control	1.464	1508	1.432	1.436
Treatment	1.550	1.553	1.476	1.491
*p*-value	0.031	0.258	0.272	0.1605

**Table 4:** Analysis of the composition of the control diet, done with the portable NIR instrument Polispec.

Parameter	December	January	February	March	April
d.m. ^1^, %	54.91	54.95	54.85	54.65	54.70
Ash, % d.m.	9.20	9.76	9.69	9.23	9,76
Crude protein, % d.m.	15.96	16.21	16.20	15.83	16.17
Fats, % d.m.	3.00	2.99	3.15	3.00	3.02
NDF, % d.m.	36.35	36.65	36.20	36.43	36,68
Cellulose, % d.m.	18.74	18.77	19.27	18.82	18,71
Lignin, % d.m.	3.92	3.89	3.89	3.93	3.94
Hemicellulose, % d.m.	13.70	14.00	13.05	13.68	14.03
Starch, % d.m.	28.00	28.15	28.07	28.15	28.19
Sugars and pectins, % d.m.	7.49	6.25	6.70	7.37	6.20

^1^ d.m. = dry matter.

**Table 5:** Analysis of the composition of the Treatment diet, done with the portable NIR instrument Polispec.

Parameter	December	January	February	March	April
d.m. ^1^, %	53.79	53.89	53.78	54.63	54.55
Ash, % d.m.	9.10	9.80	9.55	9.19	9.72
Crude protein, % d.m.	16.00	16.19	16.35	15.86	16.05
Fats, % d.m.	2.88	3.05	3.05	3.00	3.03
NDF, % d.m.	36.55	36.25	36.10	36.55	36.80
Cellulose, % d.m.	18.80	18.46	18.44	18.82	18.73
Lignin, % d.m.	3.85	3.95	3.90	3.93	3.89
Hemicellulose, % d.m.	13.90	13.85	13.77	13.80	14.17
Starch, % d.m.	28.38	28.42	28.25	28.05	28.21
Sugars and pectins, % d.m.	7.10	6.30	6.70	7.35	6.20

^1^ d.m. = dry matter.

**Table 6:** Analysis of the composition of the Control feces, done with the portable NIR instrument Polispec.

Parameter	December	January	February	March	April
Moisture, %	86.97	86.88	86.60	86.90	87.03
Dry matter, %	13.03	13.13	13.40	13.10	12.97
Ash, % d.m.	9.27	9.47	8.97	9.38	9.25
Crude protein, % d.m.	17.00	17.05	17.15	17.27	17.00
Fats, % d.m.	2.75	2.55	2.57	2.78	2.64
NDF, % d.m.	61.65	61.63	61.50	61.47	61.67
Cellulose, % d.m.	35.93	36.28	35.30	35.70	35.81
Lignin, % d.m.	11.21	11.12	11.25	11.28	11.37
Hemicellulose, % d.m.	14.51	14.23	14.95	14.57	14.50
Starch, % d.m.	5.90	5.91	6.11	5.68	6.03
Sugars and pectins, % d.m.	2.44	2.40	2.71	2.42	2.41

d.m. = dry matter.

**Table 7:** Analysis of the composition of the Treatment feces, done with the portable NIR instrument Polispec.

Parameter	December	January	February	March	April
Moisture, %	86.84	87.25	86.00	86.90	87.03
d.m., %	13.16	12.75	14.00	13.10	12.96
Ash, % d.m.	9.52	10.25	9.25	10.03	10.03
Crude protein, % d.m.	16.46	16.45	16.68	16.87	16.60
Fats, % d.m.	2.70	2.89	2.63	2.85	2.82
NDF, % d.m.	62.12	60.76	61.70	60.52	60.91
Cellulose, % d.m.	35.75	34.85	34.26	34.22	35.01
Lignin, % d.m.	11.35	11.58	11.93	11.28	11.37
Hemicellulose, % d.m.	14.93	14.34	15.42	15.02	14.53
Starch, % d.m.	5.80	6.25	6.00	6.20	6.18
Sugars and pectins, % d.m.	2.40	2.41	2.75	2.54	2.47

d.m. = dry matter.

**Table 8:** Digestibility in the two groups.

Month	December	January	February	March	April	Average	P(g) ^1^	P(m) ^1^	P(g * m) ^1^
Group	**Ash, %**								
Control	64.81	66.11	67.92	64.56	67.14	66.10	0.069	0.010	0.528
Treatment	64.52	64.36	68.35	61.91	64.68	64.77			
*Sem*	*1.04*	*1.04*	*1.04*	*1.04*	*1.04*	*0.46*			
*p-*value	*0.848*	*0.264*	*0.775*	*0.103*	*0.127*	*0.069*			
	**Crude Protein, %**								
Control	62.79	63.25	63.42	61.97	63.56	63.00	0.012	0.201	0.763
Treatment	65.06	65.37	66.66	62.90	64.59	64.92			
*Sem*	*0.99*	*0.99*	*0.99*	*0.99*	*0.99*	*0.44*			
*p-*value	*0.139*	*0.164*	*0.044*	*0.524*	*0.483*	*0.012*			
	**Fats, %**								
Control	66.48	70.27	71.94	67.86	69.42	69.20	0.621	0.018	0.324
Treatment	69.20	68.02	72.32	65.36	68.91	68.76			
*Sem*	*1.33*	*1.34*	*1.34*	*1.34*	*1.34*	*0.60*			
*p-*value	*0.184*	*0.266*	*0.848*	*0.218*	*0.794*	*0.621*			
	**NDF ^2^, %**								
Control	40.77	41.27	41.29	41.18	41.71	41.24	0.039	0.821	0.952
Treatment	41.32	42.89	44.16	42.26	43.32	42.99			
*Sem*	*1.16*	*1.16*	*1.16*	*1.16*	*1.16*	*1.16*			
*p-*value	*0.368*	*0.349*	*0.113*	*0.528*	*0.351*	*0.039*			
	**Cellulose, %**								
Control	33.02	32.47	36.59	33.88	33.66	33.93	0.033	0.234	0.999
Treatment	35.52	35.64	39.30	36.57	36.01	36.61			
*Sem*	*1.71*	*1.71*	*1.71*	*1.71*	*1.71*	*1.71*			
*p-*value	*0.329*	*0.221*	*0.292*	*0.295*	*0.357*	*0.033*			
	**Hemicellulose, %**								
Control	63.00	64.52	60.39	62.87	64.17	62.99	0.470	0.343	0.783
Treatment	63.47	64.76	63.41	62.04	64.88	63.71			
*Sem*	*1.54*	*1.54*	*1.54*	*1.54*	*1.54*	*2.76*			
*p-*value	*0.833*	*0.911*	*0.193*	*0.710*	*0.746*	*0.470*			
	**Starch, %**								
Control	92.64	92.66	92.47	92.97	92.58	92.66	0.874	0.456	0.045
Treatment	93.07	92.50	93.06	92.29	92.50	92.68			
*Sem*	*0.18*	*0.18*	*0.18*	*0.18*	*0.18*	*0.08*			
*p-*value	*0.132*	*0.550*	*0.05*	*0.028*	*0.776*	*0.874*			
	**Sugars and Pectins, %**								
Control	88.65	86.58	85.88	88.58	86.54	87.25	0.903	0.003	0.753
Treatment	88.53	87.00	86.57	87.07	86.36	87.29			
*Sem*	*0.53*	*0.53*	*0.53*	*0.53*	*0.53*	*0.23*			
*p-*value	*0.883*	*0.583*	*0.382*	*0.441*	*0.817*	*0.903*			

^1^ g = group; m = month; g * m = group * month; ^2^ NDF = neutral detergent fiber.

**Table 9:** Environmental impact: carbon footprint (CFP) and predicted enteric CH_4_ production in the two groups.

	Group		SEM	*p*-Value
	Control	Treatment		
**Diet CFP ^1^**				
CO_2_ eq ^2^ g/DMI (kg/DMI)	12484.00 (12.48)	10764.00 (10.76)	22.45 (0.22)	<0.0001
CO_2_ eq g/L milk	320.53	266.40	0.79	<0.0001
**Predicted enteric CH_4_^3^ Production**				
CH_4_, g/d	475.28	460.45	0.90	<0.0001
CH_4_, g/L milk	12.20	11.30	0.03	<0.0001

^1^ CFP = Carbon footprint of the two feeds; ^2^ CO_2_ eq = equivalent to the carbon dioxide; ^3^ CH_4_ = methane.

## Data Availability

The data presented in this study are available on request from the corresponding author.

## Supplementary Materials

The following are available online at https://www.mdpi.com/article/10.3390/ani11082405/s1, Table S1: Technical information about the product Protigen used in the present trial: particle size, measured in millimetres, and in vitro release rate at different time point.
