# Supplementary material for: Effect of Slow-Release Urea Administration on Production Performance, Health Status, Diet Digestibility, and Environmental Sustainability in Lactating Dairy Cows
Source: Animals (Basel). 2021 Aug 14;11(8):2405. doi: 10.3390/ani11082405 (PMC8388657; doi:10.3390/ani11082405)
Supplement: Supplementary file 1 [file animals-11-02405-s001.zip › animals-1303832-supplementary.pdf]

Supplementary Table S1 Technical information about the product Protigen used in the present trial: particle size, measured in millimetres, and in vitro release rate at different time point.

Parameter	Value
**Particle size, mm**	
% of the product between 1.5-2.00 mm	97
% of the product <1.5 mm	3
**Release rate, %**	
At 1h	22.3
At 2h	33.1
At 3h	42.2
At 4h	49.1
At 5h	50.4
At 6h	60.2
At 7h	70.3
At 24h	99.1
